# Elevated low-density lipoprotein cholesterol levels and prostate cancer risk: UK Biobank evidence

**DOI:** 10.1007/s00345-026-06313-4

**Published:** 2026-02-27

**Authors:** R. Alexa, J. Kranz, M. Thöne, S. Grundl, M. Hoffmann, P. H. Koop, C. Feng, K. M. Schneider, C. Schneider, M. Saar

**Affiliations:** 1 Department of Urology and Pediatric Urology, University Hospital, RWTH Aachen University, Aachen, Germany; 2 Center for Integrated Oncology, Aachen Bonn Cologne Düsseldorf, Aachen, Germany; 3 Medical Clinic III, Gastroenterology, Metabolic diseases and Intensive Care, RWTH Aachen University, Aachen, Germany; 4 Department of General, Visceral, Pediatric and Transplantation Surgery, RWTH Aachen University, Aachen, Germany

**Keywords:** LDL, ALT, Diabetes mellitus, Genetic factors, Cardiovascular disease

## Abstract

**Purpose:**

Hepatic lipid metabolism has been implicated in cancer development, with cholesterol dysregulation linked to tumor initiation and progression. We examined the association between low-density lipoprotein cholesterol (LDL) levels and prostate cancer (PCa) risk in the UK Biobank cohort.

**Methods:**

This multicenter, community-based cohort study, conducted from March 2006 to December 2010, included 502,511 participants from the UK Biobank. Morbidity and mortality were analyzed in August 2023. Hazard ratios (HRs) for PCa related to hepatic blood value changes were calculated, adjusting for key variables.

**Results:**

We extracted 204,403 healthy men and 13,205 PCa patients, subdivided into 3751 with PCa at inclusion and 9454 who developed PCa during 14.1 years of follow-up. Elevated LDL ≥ 3.65 mmol/L was associated with a reduced risk of PCa (HR = 0.83, 95% CI = 0.77–0.90, *p* < 0.01). To exclude confounding from cardiovascular morbidity, we compared the prevalence of myocardial infarction, stroke, and peripheral arterial disease between LDL groups. The combined prevalence of myocardial infarction, stroke, or peripheral arterial disease was similar between low and high LDL (2.67% vs. 2.49%; OR = 0.93, *p* < 0.01). Fine–Gray competing risk models further confirmed that high LDL remained inversely associated with PCa risk (sHR = 0.92, 95% CI = 0.88–0.96, *p* < 0.01), indicating that the observed association is not explained by excess cardiovascular mortality or differential cardiovascular morbidity. A significant association was also found between paternal PCa and the risk of PCa (HR = 1.34, 95% CI = 1.16–1.54, *p* < 0.01). Elevated alanine aminotransferase (ALT ≥ 50 U/L) was protective (HR = 0.69, 95% CI = 0.56–0.84, *p* < 0.01), and matched cohort analyses confirmed a protective association with diabetes mellitus (HR = 0.78, 95% CI = 0.68–0.90, *p* < 0.01).

**Conclusion:**

Elevated LDL was consistently associated with a reduced risk of PCa, independent of cardiovascular morbidity and competing mortality. ALT elevation and diabetes mellitus also showed protective associations, though less pronounced.

**Supplementary Information:**

The online version contains supplementary material available at 10.1007/s00345-026-06313-4.

## Introduction

Globally, prostate cancer (PCa) is the second most prevalent solid tumor amongst men [1]. The association between hepatic function and PCa involves a complex interplay of biological processes, particularly when considering the impact of liver metastasis and hormonal therapy on PCa progression and treatment outcomes [2–3].

Despite this known association, research directly examining how hepatic function influences PCa risk remains sparse. Indirect evidence largely comes from studies on metabolic syndrome and its components. Obesity, for example, contributes to PCa risk through proinflammatory mediators, increased leptin, and reduced adiponectin, all of which promote metabolic disturbances such as type 2 diabetes and dyslipidemia [4–9].

Given the close link between obesity and LDL metabolism, it is noteworthy that large observational cohorts have paradoxically shown that very low LDL levels are associated with an increased incidence of PCa—an effect inconsistent with the notion that “lower is always better” [10]. In parallel, seminal in vitro studies demonstrate that PCa cells, particularly androgen-independent sublines, upregulate cholesterol uptake and depend on extracellular LDL to sustain proliferation and survival [11]. This apparent “lipid paradox” contrasts lower systemic LDL levels with higher PCa incidence, despite cholesterol being a known metabolic substrate for tumor growth, underscoring a discrepancy between population-level associations and cellular-level mechanisms.

Based on the apparent discrepancy between experimental evidence showing cholesterol dependency of PCa cells and epidemiological observations suggesting increased PCa risk at very low LDL levels, we hypothesized that systemic LDL concentrations and hepatic metabolic markers may be inversely associated with PCa incidence at the population level. We further hypothesized that this association is independent of cardiovascular morbidity and competing mortality. To test this, we leveraged the UK Biobank to examine the relationship between lipid metabolism—as reflected by hepatic blood values—and PCa risk in a large population-based cohort.

## Patients and methods

### UKB participants

The UKB is a population-based cohort study conducted in the UK from March 2006 to December 2010 that recruited 502,511 volunteers aged 37 to 73 years at baseline. All participants gave written informed consent for genotyping and data linkage to medical reports, were registered with the UK National Health Service and attended an initial examination, which was followed by a long-term follow-up that takes place continuously [12].

**Fig. 1:**
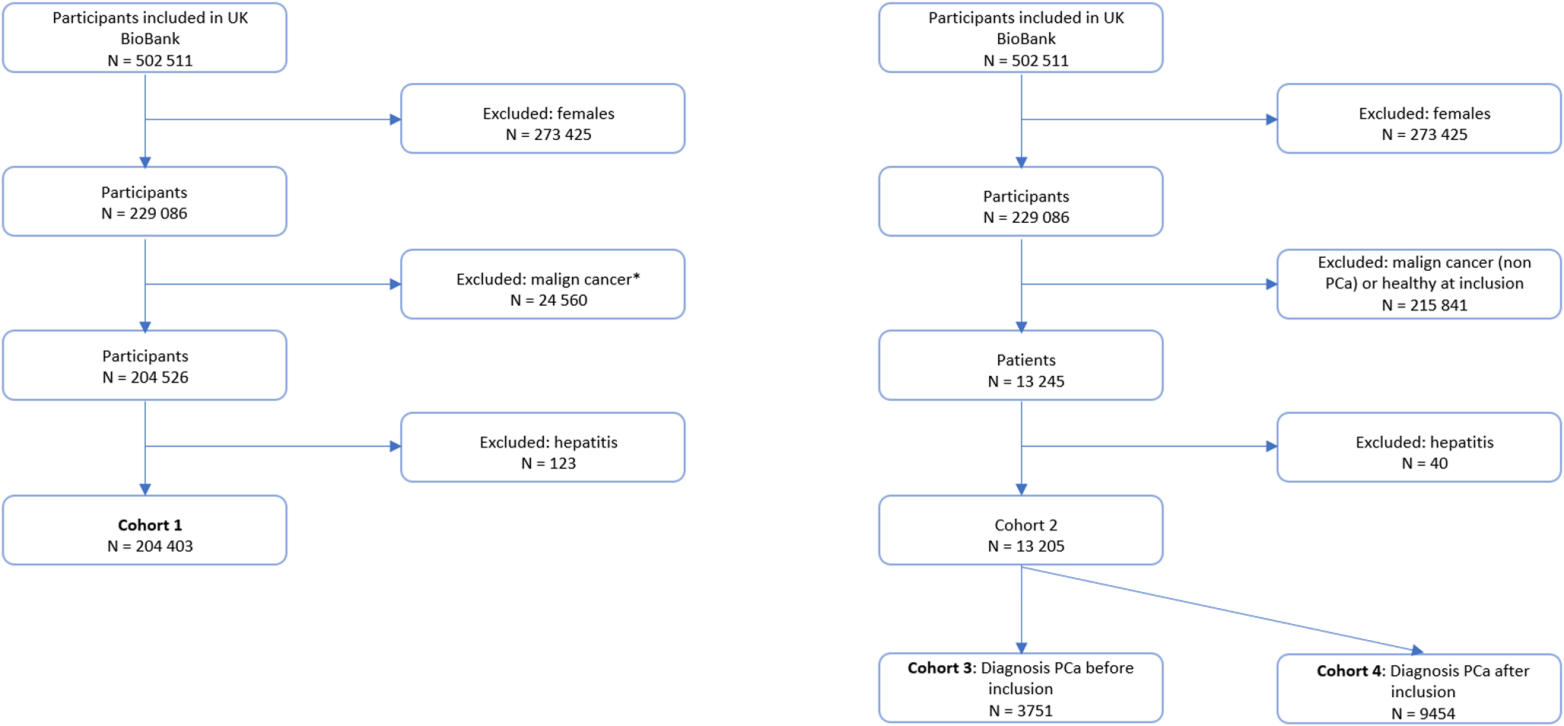
Flowchart of patient inclusion. *Diagnosis at the time of blood sampling and inclusion in the study. End of follow-up was defined as death or end of hospital inpatient data collection in August 2023

Ongoing inpatient hospital records from 1996 until August 2023 were used to identify diagnoses according to the International Statistical Classification of Diseases and Related Health Problems, Tenth Revision (ICD-10) codes. The main cohorts of the analysis are shown in Fig. 1.

### Statistical analysis

The primary outcomes of this study were the incidence of PCa and the identification of risk factors for PCa development. In addition to LDL as the primary exposure, we included secondary metabolic covariates—alanine aminotransferase (ALT), aspartate aminotransferase (AST), and alkaline phosphatase (ALP)—as well as diabetes mellitus and the self-rated health index, based on prior evidence linking hepatic function, and metabolic health to PCa risk and detection; these analyses were considered exploratory and complementary to the primary LDL-focused analyses. Genetic risk was additionally approximated using family history variables (paternal PCa and maternal breast cancer). The primary analyses were further adjusted for age, ethnicity and objectively measured body mass index (BMI; calculated as weight in kilograms divided by the square of height in meters) at baseline.

### Data and code availability

The data sets used in this study have not been deposited in a public repository but are available after approval of a reasonable application at https://www.ukbiobank.ac.uk. All participants provided written informed consent for the study. The UKB study was approved by the Northwest Multicenter research ethics committee (project 71300).

### Statistical tests

The Kolmogorov–Smirnov test was used to compare empirical distributions between groups. Cox proportional hazards regression assessed the impact of multiple cofactors on time-to-event data, with model assumptions (proportionality of hazards) verified through diagnostic tests. Logistic regression was used for binary outcomes, and propensity score matching was conducted as a sensitivity analysis to assess robustness of the primary multivariable time-to-event models. Restricted cubic spline plots explored the association between estimated LDL and PCa risk [13]. The spline analysis was performed for descriptive visualization of the continuous association between LDL levels and PCa risk and was considered complementary to the primary time-to-event analyses. Statistical significance was set at *p* < 0.01, and results were reported as hazard ratios (HRs) or odds ratios (ORs) with 95% CIs. All analyses were performed using Python.

### Cardiovascular comorbidities and competing risk analysis

Myocardial infarction (ICD-10 I21–I22), stroke (ICD-10 I60–I64), and peripheral arterial disease (ICD-10 I70 and I73) were identified from ICD-10 summary fields. Their prevalence by LDL category (< 3.65 vs. ≥ 3.65 mmol/L) was compared using logistic regression (ORs, 95% CI). Competing risk analyses used Fine–Gray models (event: PCa; competing event: death), with Gray’s test for cumulative incidence functions (CIFs). Absolute 5- and 10-year PCa risks were derived from CIF estimates.

## Results

Among the 13,245 patients with PCa a total of 9454 men developed PCa during a median follow-up time of 14.11 years. Participant characteristics cohort 1 (204403 participants) and cohort 4 (men who developed PCa after inclusion) are shown in Online Resource 1. When assessing hepatic function between cohort 1 (control group) and cohort 4 (men who developed PCa after inclusion), the Kolmogorov-Smirnov Test indicated statistically significant differences in almost all measured biomarkers, although the absolute value differences were small (additional data are given in Online Resource 2).

**Fig. 2:**
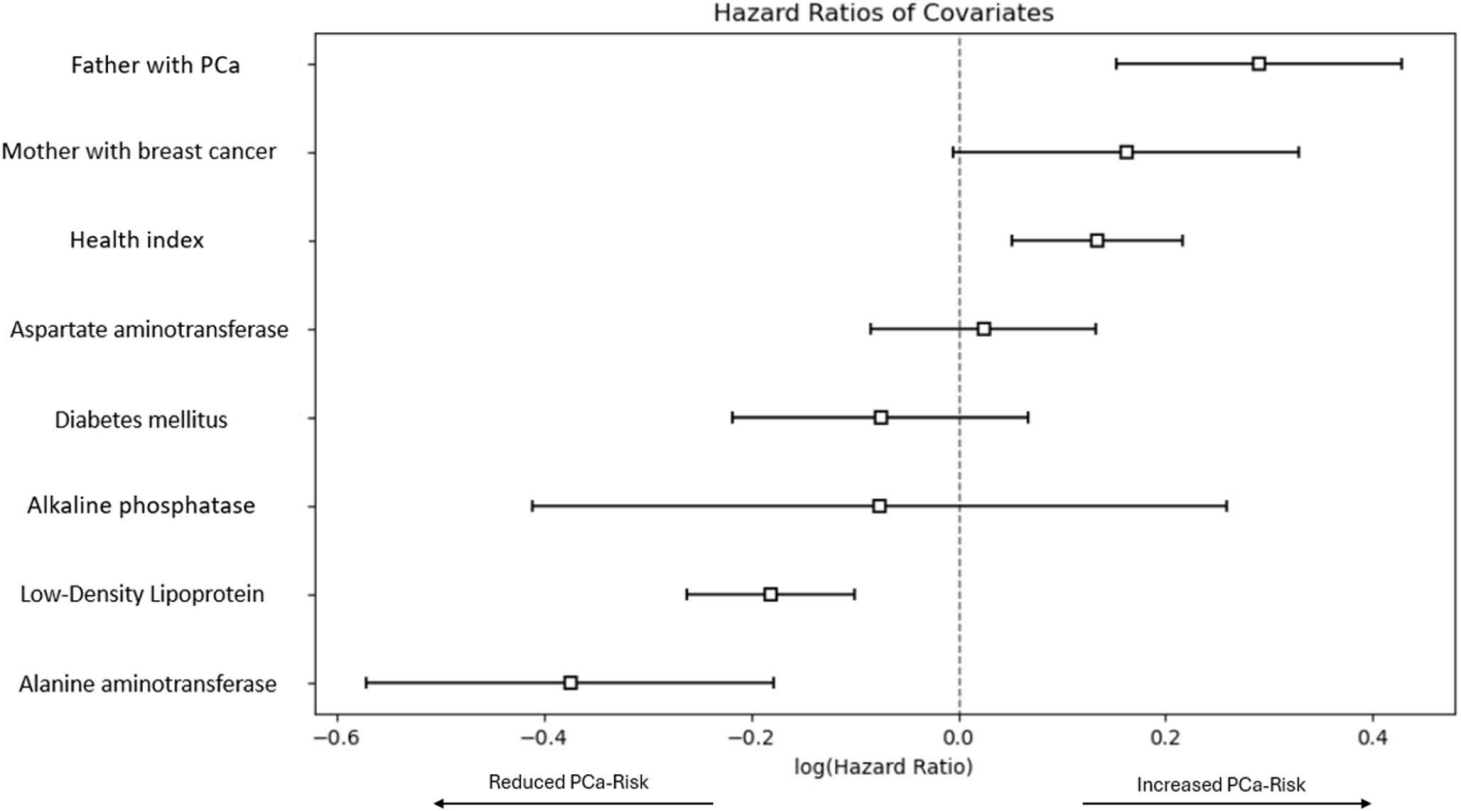
Cox regression model for PCa Risk for cohort 1 and cohort 4 (additional data are given in Online Resource 3)

To analyze associations between hepatic function, genetic factors, health index, diabetes mellitus and the risk of PCa we performed a multivariable cox proportional hazards regression between cohort 1 (control group) and cohort 4 (patients that developed PCA) (see Fig. 2).

Significant associations were found between the presence of PCa in the father, a good health index, and the risk of PCa (HR = 1.34, 95%CI = 1.16–1.54, *p* < 0.01; HR = 1.14, 95%CI = 1.05–1.24, *p* < 0.01). A protective effect was observed with an elevated LDL ≥ 3.65 mmol/L and ALT ≥ 50 U/L (HR = 0.83, 95%CI = 0.77–0.90, *p* < 0.01; HR = 0.69, 95%CI = 0.56–0.84, *p* < 0.01).

We conducted a sensitivity analysis using logistic regression to compare cohort 1 (control group) with cohort 3 (patients diagnosed with PCa at the time of inclusion). In this model, each unit increase in ALT (alanine aminotransferase) was associated with a 2% reduction in the odds of PCa (OR = 0.98, 95% CI = 0.98–0.99; *p* < 0.01). Similarly, each unit increase in LDL direct corresponded to a 10% reduction in PCa odds (OR = 0.90, 95% CI = 0.86–0.94; *p* < 0.01), demonstrating a significant protective association.

Due to the cohort’s number discrepancy, we performed a matching 5:1 based on age, ethnicity and BMI between cohort 1 and 4 and afterwards performed a cox regression model (cohorts characteristics are given Online Resource 4 and 5). In this analysis a significant association was still found between the presence of PCa in the father and the risk of PCa (HR = 1.41, 95% CI = 1.22–1.61, *p* < 0.01). A protective effect was observed with the presence of diabetes mellitus (HR = 0.78, 95% CI = 0.68–0.90, *p* < 0.01), while elevated LDL levels (≥ 3.65 mmol/L) also suggested a protective direction (HR = 0.94, 95% CI = 0.87–1.02, *p* > 0.01), though this did not reach conventional statistical significance (additional data are given in Online Resource 6).

**Fig. 3:**
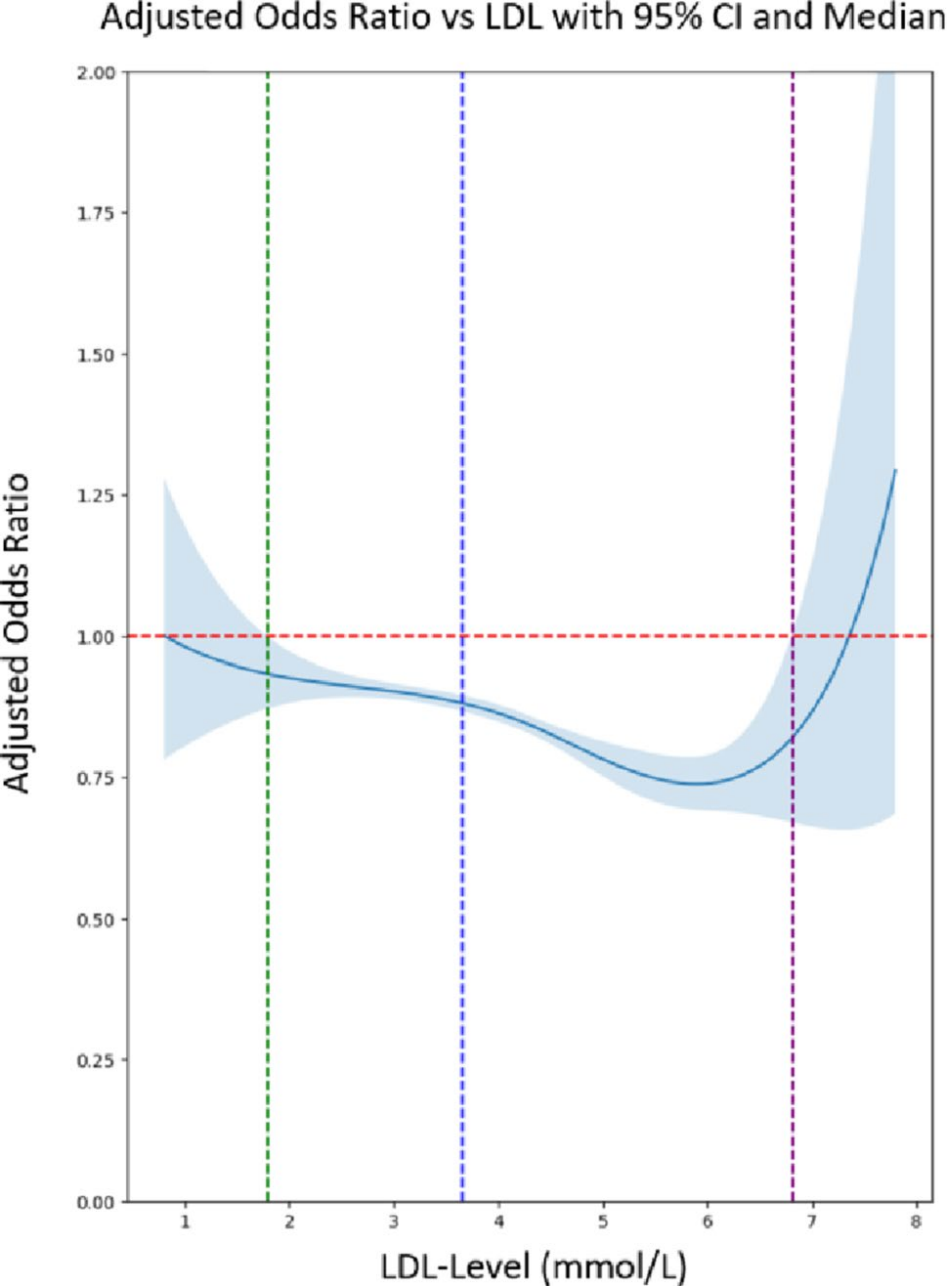
Relationship between LDL levels in mmol/L and the adjusted odds ratio of developing PCa (cohort 1 and cohort 4); LDL = 1.79 mmol/L (green line) – level where the analysis becomes statistically significant (protective effect starts); LDL = 3.65 mmol/L (blue line) – fixed reference value representing the higher upper normal limit; LDL = 6.80 mmol/L (purple line) – level where the analysis becomes statistically insignificant again. Models were adjusted for age, BMI, and ethnicity

For a deeper understanding of the level of LDL and the risk of PCa we generated a spline curve (s. Figure 3). The spline regression analysis portrays the adjusted odds ratios for developing PCa in relation to LDL, with confidence intervals illustrating the precision of the estimates. The LDL spline curve reveals a uniform trend, where between 1.79 and 6.8 mmol/L, increasing LDL appears to be protective against PCa.

### Cardiovascular comorbidities and competing risk analysis

In total, 2.67% of men with LDL < 3.65 mmol/L and 2.49% of men with LDL ≥ 3.65 mmol/L had a history of myocardial infarction (MI), stroke, or peripheral arterial disease (pAVD) (OR = 0.93, 95% CI = 0.88–0.98, *p* < 0.01). The prevalence of MI was comparable between LDL groups (1.71% vs. 1.67%; OR = 0.98, 95%CI 0.92–1.05, *p* > 0.01), whereas stroke was slightly less frequent in men with high LDL (0.68% vs. 0.79%; OR = 0.85, 95% CI = 0.77–0.95, *p* < 0.01). The peripheral arterial disease was also rare and showed no difference between LDL groups (0.14% vs. 0.18%; OR = 0.81, 95% CI = 0.65–1.01, *p* > 0.01). Competing risk analyses demonstrated that the cumulative incidence of PCa differed significantly between LDL groups (Gray’s test *p* < 0.01). In models with death as a competing event, high LDL was associated with a lower risk of PCa diagnosis (sHR = 0.92, 95% CI = 0.88–0.96, *p* < 0.01) (additional data are given in Online Resource 7).

## Discussion

In our large community-based cohort of 229,086 men, we found that elevated LDL was consistently associated with a reduced risk of developing PCa. We also observed protective associations for elevated ALT and for diabetes mellitus, although these effects were less pronounced. In addition, we reaffirmed the influence of established genetic risk factors for PCa.

### LDL and risk of PCa

Cholesterol is an essential component of cellular membranes, critical for the growth of cancer cells [12]. While there is a noted clinical correlation between hypercholesterolemia and the progression of PCa, the precise role of extracellular LDL in this context remains unclear [14–16].

In vitro studies have shown that extracellular lipid levels and LDL availability support the growth of androgen-independent PCa cells, yet observational data suggest the opposite: PCa risk increases once blood LDL drops below approximately 1.73 mmol/L (67 mg/dL), indicating that lower LDL is not always better in clinical populations [10–11]. Building on this, our findings reveal a similarly intriguing pattern: higher LDL levels appeared to exert a protective effect on the development of PCa (cohort 1 vs. cohort 4; HR = 0.83, 95% CI = 0.77–0.90, *p* < 0.01). After matching the cohorts, elevated LDL ≥ 3.65 mmol/L continued to point in the same direction (HR = 0.94, 95% CI = 0.87–1.02, *p* > 0.01), although this did not meet conventional levels of statistical significance. Our sensitivity analysis using logistic regression produced consistent results: the model showed that each unit increase in LDL was associated with a 10% reduction in the odds of PCa (OR = 0.90, 95% CI 0.86–0.94; *p* < 0.01), thereby reinforcing the protective association observed in our cox models. Importantly, we tested whether the observed association could be explained by competing risks from cardiovascular morbidity, that is, whether the prevalence of cardiovascular disease or mortality from cardiovascular causes might preclude the diagnosis of PCa. The combined prevalence of MI, stroke or pAVD was similar in both groups of high and low LDL (2.67% vs. 2.49%; OR = 0.93, *p* < 0.01). Together with Fine–Gray competing risk analyses, which confirmed that elevated LDL remained associated with a reduced subdistribution hazard of PCa diagnosis (sHR 0.92, 95%CI 0.88–0.96, *p* < 0.01), these results indicate that the apparent protective effect of LDL is not an artifact of excess cardiovascular mortality or reduced opportunity for timely PCa detection.

Mechanistically, experimental data indicate that PCa cells can sustain proliferation through enhanced intracellular cholesterol synthesis and regulated uptake pathways, suggesting that tumor-level cholesterol dependence may be partly decoupled from circulating LDL concentrations measured in population-based cohorts [11, 14]. Alternatively, low LDL may act as a marker of broader metabolic or hormonal milieus (e.g., androgen-related or inflammatory states) linked to PCa susceptibility or detection [11, 14]. Given the potentially discordant pathways linking systemic LDL levels with tumor cholesterol biology, the observed association in real-world populations may reflect a combination of biological, behavioral, and detection-related factors rather than a direct lipid-fueling effect.

### Diabetes mellitus and the risk of PCa

While many studies have found no or only weak positive links between elevated glucose levels, including type 2 diabetes mellitus, and the risk of cancer generally, the relationship appears different for PCa [17–18]. Most research in this area has identified a reverse correlation, with elevated glucose levels typically associated with a reduced risk of PCa [19]. Our analysis supports this hypothesis (cohort 1 vs. cohort 4, matched 5:1) demonstrating a protective effect of diabetes mellitus on PCa risk (HR = 0.78, 95%CI = 0.68–0.90, *p* < 0.01).

A possible explanation for this inverse relationship might be the lower insulin and insulin-like growth factor-1 (IGF-1) levels found in individuals with long-term diabetes [20]. This is relevant because high IGF-1 levels have been linked to an increased risk of PCa, especially less aggressive forms of the disease [21].

### ALT and the risk of PCa

Alanine aminotransferase (ALT) is a liver enzyme mainly involved in alanine metabolism. Low ALT has been linked to frailty and shorter survival in both healthy adults and PCa patients [22–24]. In our initial analysis, higher ALT appeared protective against PCa (HR = 0.69, 95% CI = 0.56–0.84, *p* < 0.01), and logistic regression supported this effect (OR = 0.98, 95% CI = 0.98–0.99, *p* < 0.01). However, after 5:1 matching, this association was no longer significant (HR = 0.92, 95% CI = 0.76–1.13, *p* > 0.01). Due to this inconsistency and limited supporting evidence, no further analyses of ALT were performed.

This study is subject to several limitations that warrant consideration. Firstly, we lacked detailed tumor classification, including information on grade, stage, and metastatic status. Epidemiological evidence suggests that risk factors for indolent and aggressive PCa are not identical; therefore, we cannot determine whether the associations observed in this analysis relate to clinically significant disease or predominantly to indolent tumors detected through screening. Consequently, our results should be interpreted as pertaining to overall PCa incidence and the observed association with higher LDL levels. Future studies with comprehensive tumor characterization are needed to assess whether these associations differ by disease aggressiveness.

Secondly, our dataset provides limited information regarding the therapeutic interventions and follow-up for the tumor cases studied. The absence of comprehensive treatment data, including the type, duration, and response to therapy, could hinder the ability to fully assess the impact of different treatment modalities on patient outcomes. Nonetheless, the primary aim of our study was to investigate the risk of developing PCa rather than to follow up on disease progression.

Thirdly, the observed association between a higher self-rated health index and increased PCa risk should be interpreted with caution, as self-perceived health may influence health-seeking behavior and the likelihood of undergoing diagnostic testing, thereby affecting PCa detection. Although the UK Biobank includes information on socioeconomic status, dietary patterns, and physical activity, these variables are partly self-reported and predominantly assessed at baseline with limited granularity, which may not fully capture long-term exposures. Consequently, residual confounding related to screening intensity, healthcare utilization, and other incompletely measured factors cannot be excluded.

Fourthly, detailed information on lipid-lowering therapy was not incorporated into our primary models. Statin use has been associated with reduced risks of advanced or lethal PCa in previous analyses; however, the studies conducted to date have generally had short follow-up, have not targeted clearly defined molecular subgroups, and have not provided consistent support for these observational findings [25]. Because LDL cholesterol constituted the primary exposure of interest and statin therapy both directly modifies LDL levels and is strongly linked to cardiovascular risk and healthcare utilization, adjustment for statin use could introduce over-adjustment or collider bias rather than appropriately control confounding. Nevertheless, residual confounding related to lipid-lowering therapy cannot be fully excluded.

Finally, the UK Biobank largely comprises participants of European ancestry within a UK-specific healthcare and screening environment, which may limit the generalizability of our findings to populations with different demographic compositions, risk profiles, and diagnostic practices; external validation in independent, more diverse cohorts is therefore warranted.

Thus, our results offer valuable insights and should be interpreted within the context of these constraints.

Our findings demonstrate that elevated LDL is associated with a reduced risk of PCa, even after accounting for cardiovascular morbidity and competing mortality. This paradoxical protective effect underscores the complex role of lipid metabolism in PCa biology and warrants further mechanistic investigation. The presence of diabetes mellitus and elevated ALT also showed protective associations, though less pronounced, suggesting that broader aspects of metabolic health may influence PCa risk.

## Supplementary Information

Below is the link to the electronic supplementary material.

Supplementary Material 1

Supplementary Material 2

Supplementary Material 3

Supplementary Material 4

Supplementary Material 5

Supplementary Material 6

Supplementary Material 7

## Data Availability

The data sets used in this study have not been deposited in a public repository but are available after approval of a reasonable application at https://www.ukbiobank.ac.uk/.
