# Supplementary material for: Elevated low-density lipoprotein cholesterol levels and prostate cancer risk: UK Biobank evidence
Source: World J Urol. 2026 Feb 27;44(1):210. doi: 10.1007/s00345-026-06313-4 (PMC12948794; doi:10.1007/s00345-026-06313-4)
Supplement: Supplementary file 3 — Supplementary Material 3 [file 345_2026_6313_MOESM3_ESM.pdf]

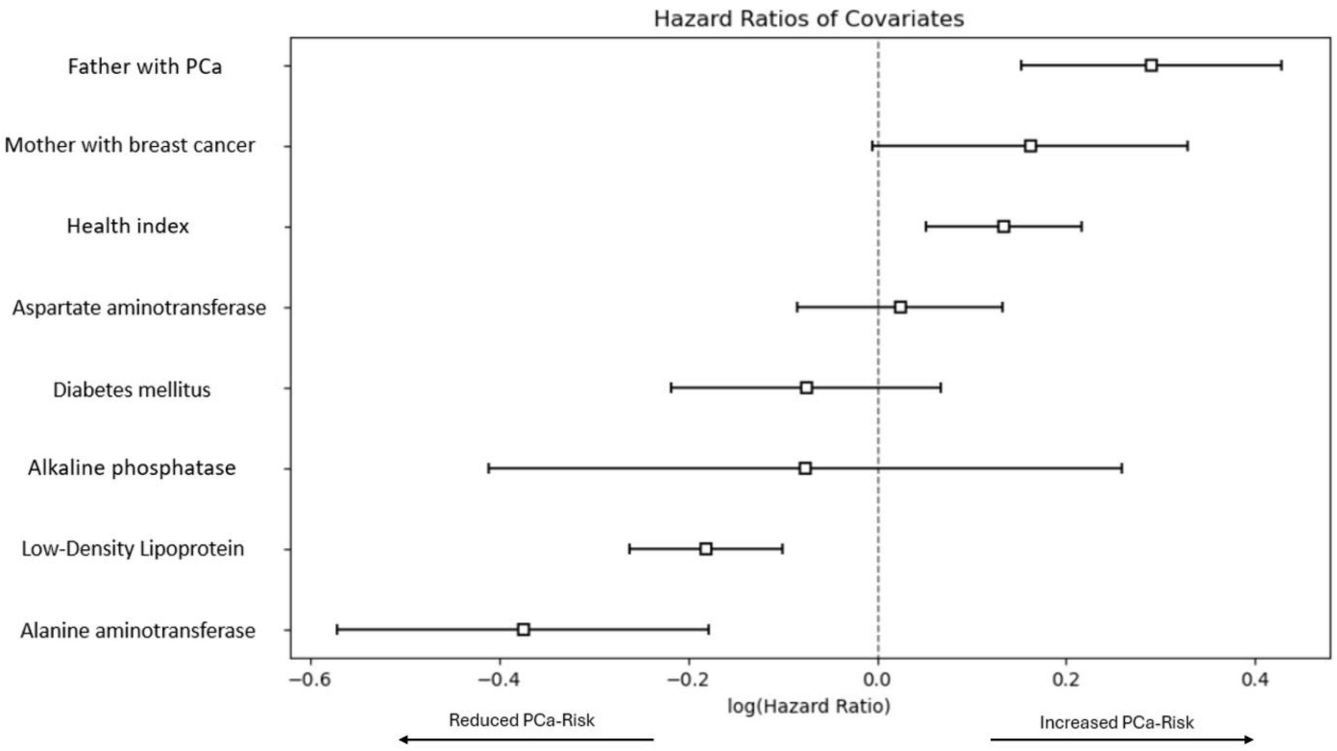

variable_name	coef	exp(coef)	se(coef)	coef_lower_95%	coef_upper_95%	exp_coef_lower_95%	exp_coef_upper_95%	z	p	-log2(p)
Father with Pca	0,29	1,34	0,07	0,15	0,43	1,16	1,54	4,12	0,00004	14,67
Mother with breast cancer	0,16	1,18	0,09	0,00	0,33	1,00	1,39	1,91	0,05666	4,14
Health index	0,13	1,14	0,04	0,05	0,22	1,05	1,24	3,18	0,00148	9,40
AST (Aspartate aminotransferase)	0,02	1,02	0,06	-0,08	0,13	0,92	1,14	0,43	0,66424	0,59
Diabetes mellitus	-0,07	0,93	0,07	-0,22	0,07	0,80	1,07	-1,03	0,30534	1,71
ALP (Alkaline phosphatase)	-0,08	0,93	0,17	-0,41	0,26	0,66	1,29	-0,45	0,65594	0,61
LDL direct	-0,18	0,83	0,04	-0,26	-0,10	0,77	0,90	-4,42	0,00001	16,63
ALT (Alanine aminotransferase)	-0,37	0,69	0,10	-0,57	-0,18	0,56	0,84	-3,73	0,00019	12,34

Online resource 3. Cox regression model for PCa Risk for cohort 1 and cohort 4
