# Supplementary material for: Elevated low-density lipoprotein cholesterol levels and prostate cancer risk: UK Biobank evidence
Source: World J Urol. 2026 Feb 27;44(1):210. doi: 10.1007/s00345-026-06313-4 (PMC12948794; doi:10.1007/s00345-026-06313-4)
Supplement: Supplementary file 6 — Supplementary Material 6 [file 345_2026_6313_MOESM6_ESM.pdf]

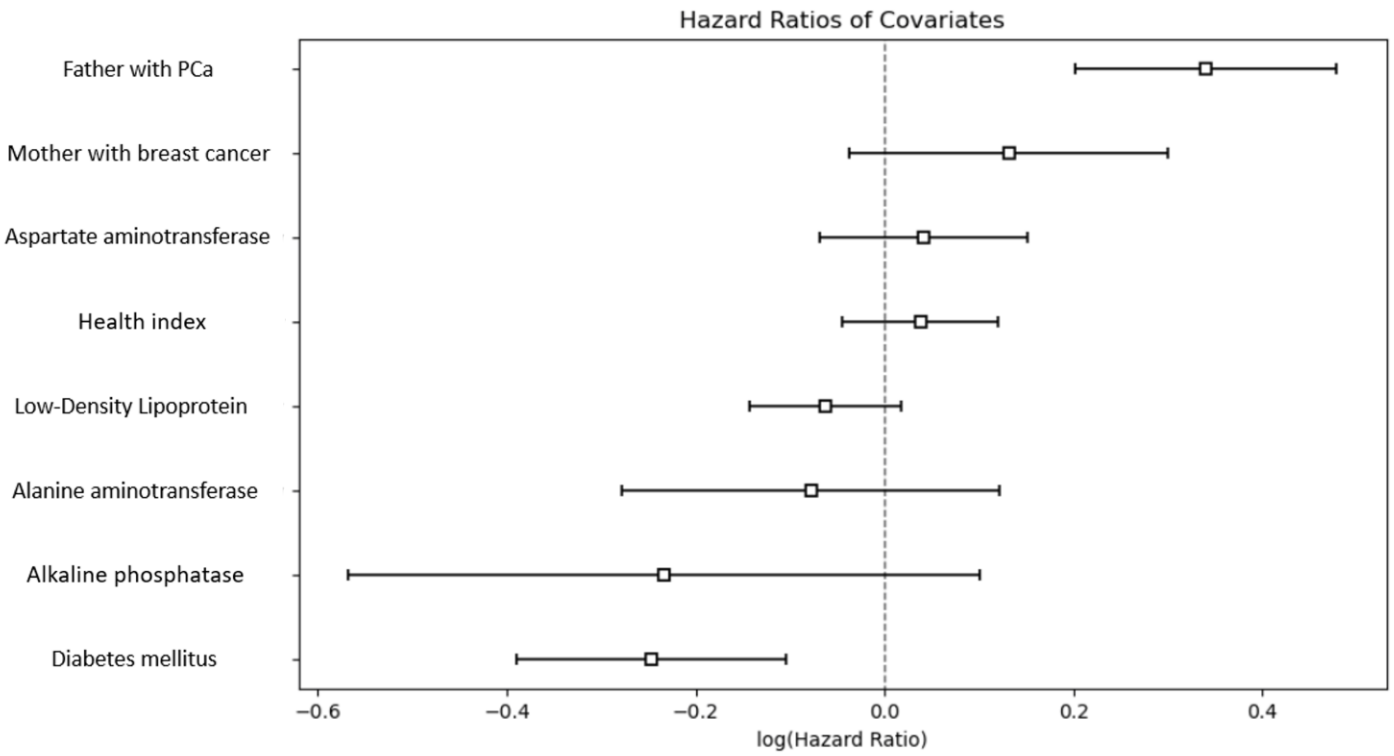

variable_name	coef	exp(coef)	se(coef)	coef_lower_95%	coef_upper_95%	exp_coef_lower_95%	exp_coef_upper_95%	z	p	-log2(p)
Father with Pca	0,34	1,41	0,07	0,20	0,48	1,22	1,61	4,82	0,000001	19,43
Mother with breast cancer	0,13	1,14	0,09	-0,04	0,30	0,96	1,35	1,53	0,125892	2,99
AST (Aspartate aminotransferase)	0,04	1,04	0,06	-0,07	0,15	0,93	1,16	0,73	0,462452	1,11
Health index	0,04	1,04	0,04	-0,05	0,12	0,96	1,13	0,89	0,373504	1,42
Low-density Lipoprotein	-0,06	0,94	0,04	-0,14	0,02	0,87	1,02	-1,54	0,124458	3,01
ALT (Alanine aminotransferase)	-0,08	0,92	0,10	-0,28	0,12	0,76	1,13	-0,77	0,441577	1,18
ALP (Alkaline phosphatase)	-0,23	0,79	0,17	-0,57	0,10	0,57	1,11	-1,37	0,170398	2,55
Diabetes mellitus	-0,25	0,78	0,07	-0,39	-0,10	0,68	0,90	-3,38	0,000717	10,45

Online resource 6. Cox regression modell after matching 1:5 (cohort 1 and cohort 4) for BMI, age and ethnicity
