# Supplementary material for: Elevated low-density lipoprotein cholesterol levels and prostate cancer risk: UK Biobank evidence
Source: World J Urol. 2026 Feb 27;44(1):210. doi: 10.1007/s00345-026-06313-4 (PMC12948794; doi:10.1007/s00345-026-06313-4)
Supplement: Supplementary file 7 — Supplementary Material 7 [file 345_2026_6313_MOESM7_ESM.pdf]

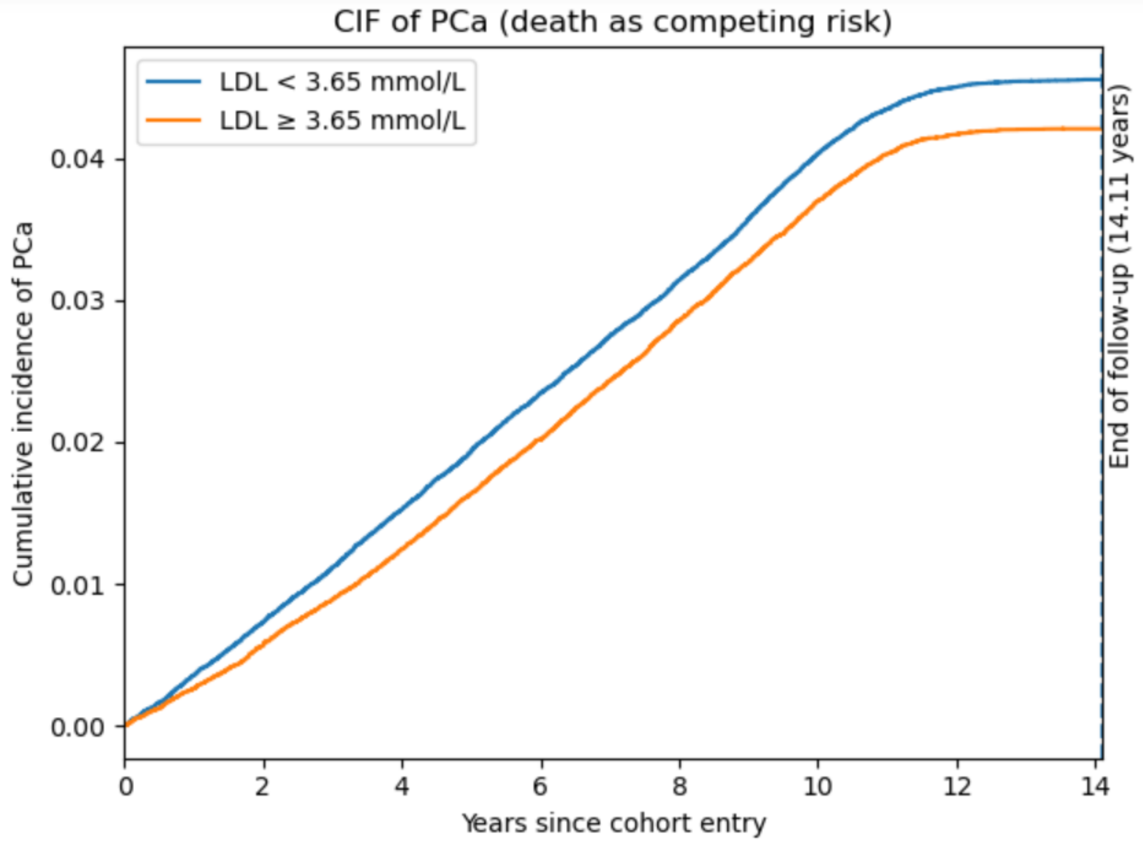

LDL group	Time (years)	CIF (absolute risk)	SE	95% CI lower	95% CI upper
< 3.65 mmol/L	5	0,019354	0,000381	0,018607	0,0201
< 3.65 mmol/L	10	0,040335	0,000544	0,039269	0,041401
≥ 3.65 mmol/L	5	0,01638	0,000441	0,015517	0,017243
≥ 3.65 mmol/L	10	0,036988	0,000655	0,035704	0,038271

Online resource 7. Cumulative incidence function (CIF) curves for prostate cancer (PCa) stratified by baseline LDL levels (< 3.65 vs. ≥ 3.65 mmol/L), accounting for death as a competing risk with 14.1 years of follow-up and absolute risk of PCa at 5 and 10 years, estimated using CIF with standard errors (SE) and 95% confidence intervals for participants with LDL < 3.65 mmol/L and ≥ 3.65 mmol/L
